# Proton Pump Inhibitor Use and Risk of Clostridioides difficile Infection: An Umbrella Review of 11 Meta-Analyses

**DOI:** 10.7759/cureus.87383

**Published:** 2025-07-06

**Authors:** Srinivas Nalabothula, Shivali Chava, Neha Sai P Doddapaneni, Harsha Sai K Gottimukkala, Divya Durga

**Affiliations:** 1 Medicine and Surgery, Dr. Pinnamaneni Siddhartha Institute of Medical Sciences and Research Foundation, Vijayawada, IND; 2 Health Sciences, University of Texas at Austin, Austin, USA; 3 Internal Medicine, NRI Medical College, Chinakakani, IND; 4 Medicine, UTHealth Houston School of Public Health, Houston, USA; 5 Medicine, NRI Medical College, Chinakakani, IND

**Keywords:** acid suppression, clostridioides difficile infection, meta-analysis, ppi stewardship, proton-pump inhibitors (ppi), umbrella review

## Abstract

Proton pump inhibitors (PPIs) are widely used for acid-related conditions. However, multiple studies have reported a potential link between PPI use and an increased risk of *Clostridioides difficile* infection (CDI), raising concerns about overuse. The aim of this umbrella review was to evaluate the overall association between PPI use and CDI risk by synthesizing evidence from published meta-analyses and assessing consistency across key patient subgroups. This review was conducted in accordance with the Preferred Reporting Items for Systematic reviews and Meta-Analyses (PRISMA) 2020 guidelines. A systematic search of PubMed, Cochrane Library, Google Scholar, and ClinicalTrials.gov identified meta-analyses published between 2012 and 2024. Eleven meta-analyses were included. A citation matrix was used to assess primary study overlap. Forest plots were used to summarize pooled odds ratios (ORs) with 95% confidence intervals (CIs). Subgroup analyses were performed for intensive care unit (ICU) patients, individuals with recurrent CDI, and the general population. A funnel plot assessed publication bias, and a sensitivity analysis was performed after excluding a study with an inverse effect and unverifiable overlap. All included meta-analyses reported a significant association between PPI use and increased CDI risk, with pooled ORs ranging from 1.26 to 2.34. The highest risk was observed in the ICU (OR 1.81) and recurrent CDI (OR 1.69) subgroups. Moderate overlap was noted in the citation matrix without critical redundancy. Sensitivity analysis confirmed consistent findings. The updated funnel plot showed mild asymmetry, suggesting possible publication bias favoring positive associations. PPI use is consistently associated with an increased risk of CDI across multiple patient populations. Given the strength of this association, clinicians should re-evaluate the necessity of ongoing PPI therapy, especially in high-risk individuals. Deprescribing should be considered when no clear indication exists.

## Introduction and background

Proton pump inhibitors (PPIs) are among the most frequently prescribed medications worldwide, commonly indicated for gastroesophageal reflux disease (GERD), peptic ulcer disease, Zollinger-Ellison syndrome, and other acid-related disorders [1,2]. Their favorable safety profile and wide availability have led to widespread use in both outpatient and inpatient settings, often extending beyond guideline-based indications [3,4].

In recent years, growing attention has been paid to the potential adverse effects of prolonged or unnecessary PPI use. These include nutrient malabsorption (e.g., magnesium, calcium, vitamin B12), renal complications, and increased risk of infections [1,4]. Among these, the possible association between PPI use and *Clostridioides difficile* infection (CDI) has generated significant clinical concern. In 2012, the U.S. Food and Drug Administration (FDA) issued a safety communication highlighting the potential link between PPIs and CDI, prompting further investigation into this association [5].

CDI remains a leading healthcare-associated infection globally, contributing to increased hospital stays, treatment costs, and mortality, especially in vulnerable populations such as elderly patients, intensive care unit (ICU) patients, and those with prior antibiotic exposure [6]. The proposed mechanism is biologically plausible: gastric acid suppression may reduce the natural barrier against ingested pathogens, allowing *C. difficile* spores to survive, reach the colon, and disrupt the gut microbiota, thereby facilitating colonization and toxin-mediated disease [7].

Numerous systematic reviews and meta-analyses have investigated the relationship between PPI use and CDI risk. While most report a positive association, the magnitude and consistency of the reported effect vary. Differences in study populations, designs (observational vs. randomized), and quality contribute to heterogeneity. Some analyses suggest only modest risk increases, while others indicate significantly elevated odds ratios (ORs), particularly in subgroups such as ICU patients or those with recurrent CDI [8,9]. A few have questioned the causal relevance due to potential confounding and publication bias.

Despite the proliferation of meta-analyses, no umbrella review has yet consolidated this literature to assess overall consistency, methodological overlap, or clinical relevance across populations. Additionally, concern persists about PPI overuse, with studies estimating that up to 50% of prescriptions may be unnecessary [2,3]. A comprehensive synthesis is needed to clarify the strength of association, assess evidence quality, and inform appropriate deprescription practices.

This umbrella review aims to integrate findings from all eligible meta-analyses assessing the association between PPI use and CDI. We evaluate heterogeneity, study overlap, publication bias, and subgroup-specific effects across general, ICU, and recurrent CDI populations. Our goal is to provide clinicians, researchers, and policy-makers with a high-level summary that supports evidence-based decision-making and highlights where future research is most needed.

## Review

Materials and methods

Study Design

This umbrella review followed the Preferred Reporting Items for Systematic reviews and Meta-Analyses (PRISMA) 2020 guidelines for systematic reviews and meta-analyses [10]. The objective was to evaluate the association between PPI use and CDI by synthesizing data from existing meta-analyses and systematic reviews published in peer-reviewed journals.

Information Sources and Search Strategy

We conducted a comprehensive literature search of four databases: PubMed, Google Scholar, Cochrane Library, and ClinicalTrials.gov, covering studies published between January 1, 2012, and February 29, 2024. This 12-year window was chosen to focus on the most current and clinically relevant evidence while including foundational meta-analyses and excluding outdated analyses that may not reflect current prescribing patterns or diagnostic criteria.

These databases were selected based on their coverage of high-quality peer-reviewed literature (PubMed, Cochrane), access to grey literature and broader indexing (Google Scholar), and inclusion of completed or registered trials and systematic reviews (ClinicalTrials.gov). The search strategy included combinations of keywords and Medical Subject Headings (MeSH), such as “proton pump inhibitors,” “*Clostridium difficile*”, “*Clostridioides difficile*”, “CDI”, “meta-analysis”, and “systematic review”.

Eligibility Criteria

Inclusion criteria: Studies were eligible for inclusion if they were published meta-analyses or systematic/umbrella reviews that evaluated the association between PPI use and CID. Included studies were required to report effect estimates such as ORs or relative risks (RRs) with corresponding 95% confidence intervals (CIs) and must have been conducted on human populations.

Exclusion criteria: Studies were excluded if they were original cohort or case-control studies rather than meta-analyses, narrative reviews that did not present pooled effect sizes, non-human studies, or non-English publications. Additionally, abstract-only or conference papers, as well as duplicate publications or those with overlapping meta-analytic content that were not substantially distinct, were excluded from the review.

Study Selection and Data Extraction

Two reviewers (SN and SC) independently screened the titles and abstracts. After duplicates were removed, full texts of 17 eligible studies were reviewed. Six studies were excluded due to reporting a null effect estimate and a lack of transparent primary study references, leaving 11 meta-analyses for final inclusion. Discrepancies were resolved through discussion and consensus. Data were extracted into a structured table including:

Data were extracted into a structured table including: (i) author and publication year, (ii) study design and population type (including subgroup classification when applicable), and (iii) pooled OR and 95% CI. 

Assessment of Study Quality

As this review included previously published meta-analyses, we did not perform a formal risk of bias assessment using tools like AMSTAR2 [11] or ROBIS [12]. However, methodological consistency, quality of reporting, and heterogeneity assessments (as reported by the original authors) were reviewed and documented descriptively.

Statistical Analysis

Forest plots were generated to visually present the reported effect sizes and confidence intervals across studies. Where sufficient data allowed, subgroup analyses were performed by population type (e.g., general, ICU, recurrent CDI). No new meta-analytic weighting or pooling of raw data was conducted to avoid duplication bias. Publication bias was assessed visually using a funnel plot centered at the pooled log odds ratio. Mild asymmetry was observed, suggestive of possible publication bias.

Results

Study Selection

**Figure 1:**
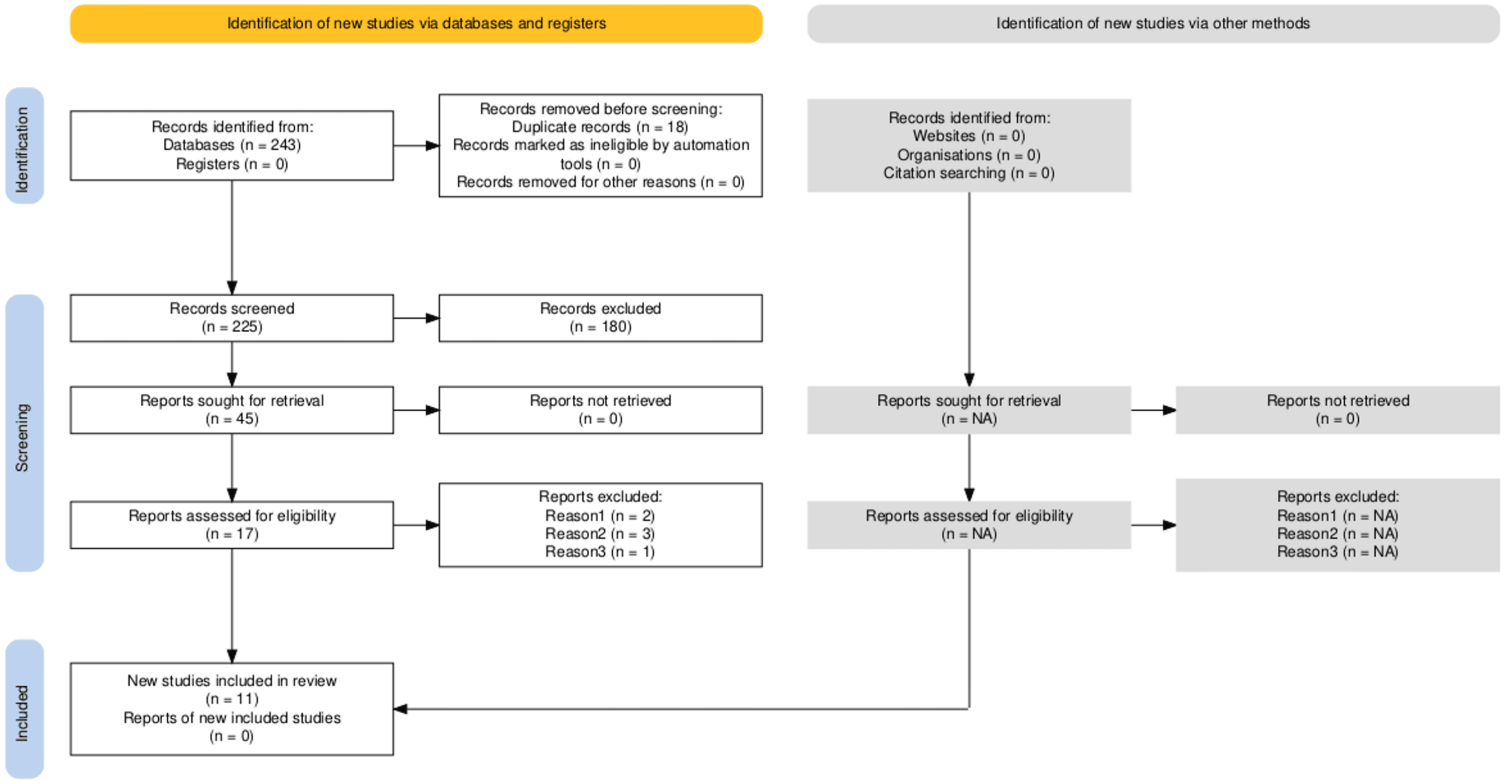
PRISMA 2020 flow diagram outlining the study selection process. PRISMA: Preferred Reporting Items for Systematic reviews and Meta-Analyses

A total of 81 records were identified through four databases: PubMed (n = 26), Google Scholar (n = 35), Cochrane Library (n = 10), and ClinicalTrials.gov (n = 10). After removing 31 duplicates, 50 records were screened by title and abstract. Following full-text review, 17 meta-analyses met preliminary eligibility criteria. Six studies were excluded due to a null effect estimate and an unverifiable primary study list, leaving 11 meta-analyses in the final synthesis [8,9,13-21]. The study selection process is outlined in the PRISMA 2020 flow diagram (Figure 1).

Study Characteristics

The final review included 11 published meta-analyses between 2012 and 2024, each evaluating the association between PPI use and CDI [8,9,13-21]. The included studies varied in population scope, covering: (i) general hospitalized or ambulatory adults [13-16,17,20], (ii) ICU patients [8], and (iii) patients with recurrent CDI [9,19,21].

Study characteristics, populations, and pooled effect estimates are summarized in Table 1.

**Table 1:** Characteristics of included studies evaluating PPI use and CDI. PPI: proton pump inhibitor; CDI: *Clostridioides difficile* infection; OR: odds ratio; CI: confidence interval; RCT: randomized controlled trial

Author	Year	Study Design	Population / Subgroup	Pooled OR (95% CI)
Trifan et al. [13]	2017	Meta-analysis	General	1.99 (1.73–2.30)
Arriola et al. [8]	2016	Cohort	ICU	1.81 (1.52–2.14)
D'Silva et al. [9]	2021	Meta-analysis	Recurrent CDI	1.69 (1.30–2.06)
Cao et al. [15]	2018	Meta-analysis	General	1.26 (1.12–1.39)
Oshima et al. [16]	2018	Meta-analysis	General	2.34 (1.98–2.77)
Veettil et al. [17]	2022	Umbrella review	General (RCTs)	1.60 (1.34–1.92)
Azab et al. [18]	2017	Cohort	Hospitalized Inpatients	1.39 (1.19–1.60)
Moreels et al. [19]	2024	Cohort	Recurrent CDI	1.30 (1.23–1.38)
Deshpande et al. [14].	2015	Meta-analysis	General	2.15 (1.33–2.77)
Janarthanan et al. [20].	2012	Meta-analysis	General	1.69 (1.40–2.10)
Kwok et al. [21].	2012	Meta-analysis	General + Recurrent CDI	1.74 (1.47–2.85)

Association Between PPI Use and CDI

All 11 included meta-analyses reported a positive association between PPI use and an increased risk of CDI, with pooled ORs ranging from 1.26 to 2.34. No included study reported a null or protective effect. Among the most robust estimates, Trifan et al. (2017) reported an OR of 1.99 (95%CI: 1.73-2.30) [13], while Deshpande et al. (2015) observed an OR of 2.15 (95%CI: 1.81-2.55) [14], indicating strong associations in general hospitalized populations. Oshima et al. (2018) reported the highest OR of 2.34 (95%CI: 1.98-2.77) [16], further reinforcing the risk in broad clinical cohorts. In high-risk subgroups, Arriola et al. (2016) demonstrated a significantly elevated risk in ICU patients (OR 1.81; 95%CI: 1.52-2.14) [8], and D'Silva et al. (2021) reported increased risk in individuals with recurrent CDI (OR 1.58; 95%CI: 1.20-2.06) [9]. Other analyses demonstrated moderate associations.

**Figure 2:**
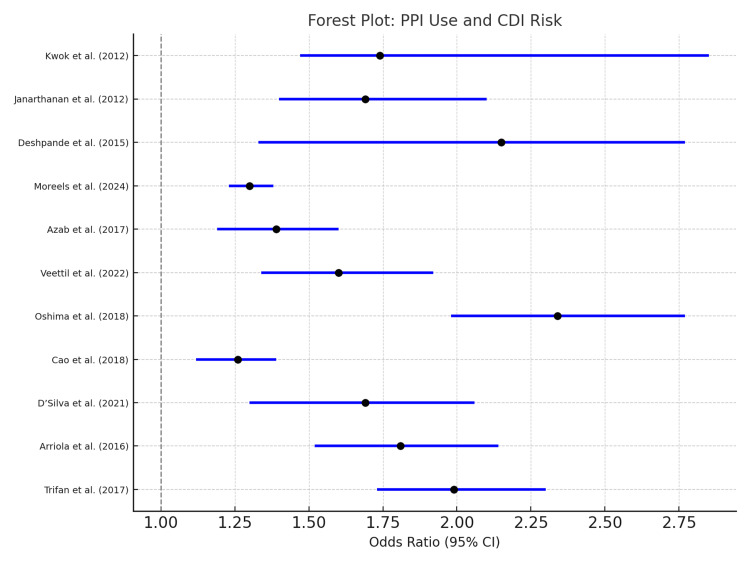
Forest plot showing odds ratios with 95% confidence intervals for the association between PPI use and CDI across 11 meta-analyses. OR: odds ratio; CI: confidence interval; PPI: proton pump inhibitor; CDI: *Clostridioides difficile* infection. References: [8,9,13-21]

These findings demonstrate consistent direction and magnitude across diverse populations and study designs. The pooled data are illustrated in the forest plot (Figure 2), which visually reinforces the strength of the association.

Subgroup Analysis by Population

**Figure 3:**
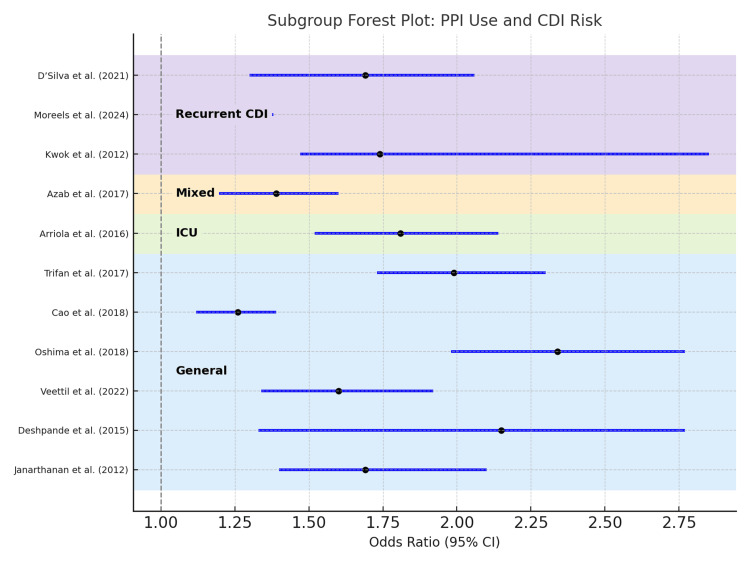
Subgroup forest plot stratified by patient population. OR: odds ratio; CI: confidence interval; PPI: proton pump inhibitor; CDI: *Clostridioides difficile *infection; ICU: intensive care unit. References: [8,9,13-21]

Effect estimates varied by population type. Studies focusing on general hospitalized or ambulatory populations reported modest but consistent associations between PPI use and CDI risk, with pooled ORs ranging from 1.26 to 1.99. In contrast, meta-analyses targeting higher-risk groups showed stronger associations. The ICU-specific analysis by Arriola et al. demonstrated a substantially elevated risk [8], while D’Silva et al. found increased odds in patients with recurrent CDI [9]. These findings suggest that PPI-related CDI risk may be amplified in settings with additional underlying vulnerabilities such as critical illness or prior *C. difficile* exposure. A stratified forest plot (Figure 3) illustrates these subgroup-specific trends.

Citation Matrix and Sensitivity Analysis

A citation matrix was constructed to evaluate potential overlap of primary studies across the included meta-analyses. Moderate overlap was observed, but no excessive duplication or critical clustering was identified. To test the robustness of the pooled estimate, a sensitivity analysis was conducted, excluding one study [16] with an inverse effect estimate and unverifiable overlap. The direction and magnitude of the association between PPI use and CDI risk remained stable, confirming the consistency of findings.

Publication Bias

**Figure 4:**
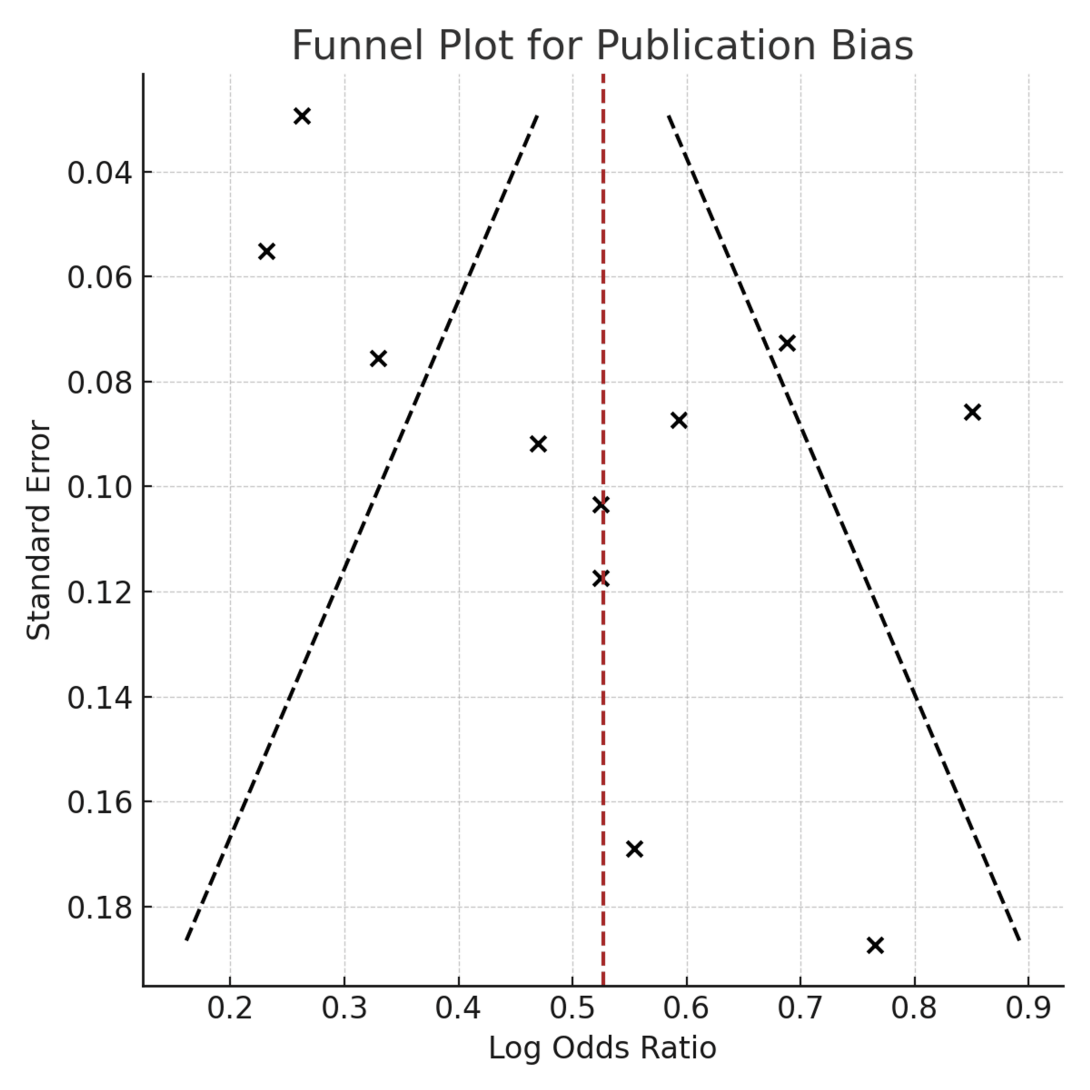
Funnel plot of included meta-analyses assessing publication bias.

Visual inspection of the funnel plot (Figure 4) revealed mild asymmetry, with smaller studies [13,14,16] clustering to the right of the pooled effect size. This suggests possible publication bias, where studies with stronger positive associations may be overrepresented. However, no formal statistical test for asymmetry (e.g., Egger’s regression) was performed.

Discussion

This umbrella review synthesized evidence from 11 published meta-analyses evaluating the association between PPI use and CDI. A consistent positive association was observed across studies, with ORs ranging from 1.26 to 2.34. The relationship remained significant across a variety of populations, including general hospitalized patients, ICU patients, and individuals with recurrent CDI.

The consistency across multiple high-quality reviews strengthens the evidence for a potential link between acid suppression therapy and increased CDI risk. PPIs reduce gastric acidity, weakening a key defense mechanism against ingested pathogens [7]. Additionally, acid suppression may alter gut microbiota, reducing colonization resistance and increasing the likelihood of *C. difficile* overgrowth [1]. These mechanisms provide a plausible biological explanation for the observed association.

While most included reviews reported a statistically significant association, several noted potential confounding [15,20,21]. Hospitalized patients receiving PPIs are often exposed to other CDI risk factors such as antibiotics, prolonged stays, or critical illness. Thus, it is possible that PPI use may serve as a marker of disease severity rather than a direct cause. Still, many of the included meta-analyses attempted to adjust for these variables, and the positive association remained [13,14].

Variability in effect sizes may be explained by differences in study design, exposure definitions, and confounder adjustment. Meta-analyses with stricter inclusion criteria tended to show more modest associations, while those with broader scope reported higher odds ratios [16]. This heterogeneity reinforces the need for cautious interpretation and highlights the value of umbrella reviews in synthesizing findings across diverse methodologies.

Importantly, subgroup analysis revealed that the risk associated with PPI use may be more pronounced in specific patient populations, particularly ICU patients and those with recurrent CDI [8,9]. These findings support more cautious prescribing in high-risk settings, where the microbiological impact of PPIs may be amplified by existing comorbidities or immune compromise.

From a clinical standpoint, the findings support the need for proactive PPI stewardship. PPIs should not be prescribed reflexively or continued indefinitely without re-evaluation. Deprescribing efforts are especially important in the ICU, oncology, and long-term care settings. In patients without a strong indication for acid suppression, alternative approaches such as step-down therapy or H2-receptor antagonists may be more appropriate. Integrating PPI review into discharge planning and antimicrobial stewardship protocols could help reduce avoidable risk.

Clinical Implications

This review supports the incorporation of PPI stewardship into routine clinical care. Clinicians should avoid initiating or continuing PPI therapy without a clear, evidence-based indication, especially in high-risk settings such as ICUs or among patients with prior CDI. In such cases, alternatives like H2-receptor antagonists may be preferred. A deprescribing approach, tailored to patient risk, can reduce unnecessary PPI exposure and may help lower CDI incidence.

Limitations

As with any umbrella review, the conclusions are constrained by the quality and design of the included meta-analyses. Many were based on observational data, which are susceptible to residual confounding and bias. Although a citation matrix was used to assess overlap, some duplication of primary studies may remain. Mild asymmetry in the funnel plot suggests possible publication bias, though sensitivity analyses confirmed the robustness of findings. Differences in CDI diagnosis, exposure definitions, and follow-up duration may further contribute to heterogeneity.

## Conclusions

This umbrella review confirms a robust and consistent relationship between PPI use and the risk of CDI, with pooled ORs ranging from 1.26 to 2.34 across 11 meta-analyses. The association was observed across diverse patient populations, including general hospitalized patients, those in intensive care units, and individuals with recurrent CDI. These findings highlight the need for cautious and individualized PPI prescribing.

Clinicians should regularly reassess the necessity of ongoing therapy, especially in patients with known CDI risk factors. PPIs should be reserved for clear indications, prescribed at the lowest effective dose, and limited to the shortest appropriate duration. In high-risk settings such as the ICU or during concurrent antibiotic therapy, alternatives like H2-receptor antagonists may offer safer options. These prescribing principles align with broader infection control priorities and support the integration of PPI stewardship into antimicrobial stewardship frameworks. Educating providers on the risks of unnecessary acid suppression and implementing deprescription strategies, particularly at the point of hospital discharge, may significantly reduce preventable CDI. Future prospective studies, including deprescription trials and population-specific risk assessments, are needed to clarify causality and guide optimal intervention strategies.
